# Will Elephants Soon Disappear from West African Savannahs?

**DOI:** 10.1371/journal.pone.0020619

**Published:** 2011-06-22

**Authors:** Philippe Bouché, Iain Douglas-Hamilton, George Wittemyer, Aimé J. Nianogo, Jean-Louis Doucet, Philippe Lejeune, Cédric Vermeulen

**Affiliations:** 1 Unité de Gestion des Ressources Forestières et des Milieux Naturels, Université de Liège Gembloux Agro-Bio Tech, Gembloux, Belgium; 2 Save The Elephants, Nairobi, Kenya; 3 Department of Fish, Wildlife and Conservation Biology, Colorado State University, Fort Collins, Colorado, United States of America; 4 Programme pour l'Afrique Centrale et Occidentale (PACO), International Union for Conservation of Nature (IUCN), Ouagadougou, Burkina Faso; 5 Department of Zoology, Oxford University, Oxford, United Kingdom; Smithsonian's National Zoological Park, United States of America

## Abstract

Precipitous declines in Africa's native fauna and flora are recognized, but few comprehensive records of these changes have been compiled. Here, we present population trends for African elephants in the 6,213,000 km^2^ Sudano-Sahelian range of West and Central Africa assessed through the analysis of aerial and ground surveys conducted over the past 4 decades. These surveys are focused on the best protected areas in the region, and therefore represent the best case scenario for the northern savanna elephants. A minimum of 7,745 elephants currently inhabit the entire region, representing a minimum decline of 50% from estimates four decades ago for these protected areas. Most of the historic range is now devoid of elephants and, therefore, was not surveyed. Of the 23 surveyed elephant populations, half are estimated to number less than 200 individuals. Historically, most populations numbering less than 200 individuals in the region were extirpated within a few decades. Declines differed by region, with Central African populations experiencing much higher declines (−76%) than those in West Africa (−33%). As a result, elephants in West Africa now account for 86% of the total surveyed. Range wide, two refuge zones retain elephants, one in West and the other in Central Africa. These zones are separated by a large distance (∼900 km) of high density human land use, suggesting connectivity between the regions is permanently cut. Within each zone, however, sporadic contacts between populations remain. Retaining such connectivity should be a high priority for conservation of elephants in this region. Specific corridors designed to reduce the isolation of the surveyed populations are proposed. The strong commitment of governments, effective law enforcement to control the illegal ivory trade and the involvement of local communities and private partners are all critical to securing the future of elephants inhabiting Africa's northern savannas.

## Introduction

Overexploitation of African elephants (*Loxodonta africana*) over the past two centuries has resulted in serious range reduction, local extirpation, and large scale declines of this keystone species [1]–[4]. Declines have accelerated in the last four decades [5]–[6]. This conservation disaster has been largely over-looked in part due to the contrasting context of growing elephant populations in other regions of Africa [5]–[6]. The sub-regional differences in the status of Africa's elephants, with populations of least concern in southern Africa and threatened populations in the rest of the continent [7], perpetuate the disagreement regarding ivory trade and debates about ivory trade bans at the Convention on International Trade in Endangered Species of Wild Fauna and Flora (CITES). Characterizing the status of elephants across the continent is critical for local conservation and management authorities as well as decisions regarding international regulations of the species.

Until the 1950s, African elephants were still widely distributed across the Sudano Sahelian range [2]. Even after the 1950s some elephant populations migrated between protected areas, mainly along scattered relics of the former Sudanian savannah habitat extending from Senegal to the Nile River [2]–[4]. However, rapid human population growth and related land use changes have seriously altered this region. In particular, the West African portion of this range has long carried the largest and some of the fastest growing human population of the continent [3], [8]. Consequently many of their original habitat have been transformed into agro-pastoral areas. The Central African portion of this range has also experienced rapid human population growth and related land use changes, the major threats in this region are unregulated deforestation and road development that facilitates the penetration of hunters into former pristine habitat [2]–[4].

The ecological importance of the remaining elephant populations is well documented [2], [3], [6], [9], emphasizing the importance of the conservation of the elephants in these regions. In addition, genetic evidence suggest that the savannah elephants in West Africa may be differentiated from the Eastern and Southern African elephant populations at a level that merits special consideration, but their taxonomic status remains uncertain [10]. Despite their recognized importance, a large scale overview of elephant population trends in the northern African savannahs has never been published. Here, we analyze elephant population trends in the Sudano Sahelian range during the last 40 years, describe the geographical distribution of elephants, and suggest priorities for conservation.

## Materials and Methods

### Study area

**Figure 1:**
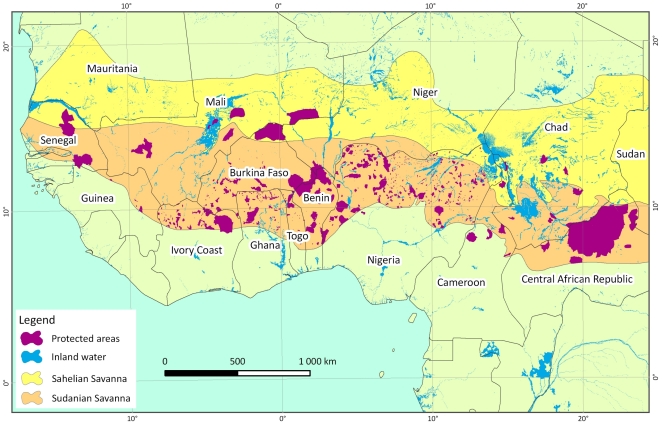
Protected areas in the Sudano Sahelian Range.

**Figure 2:**
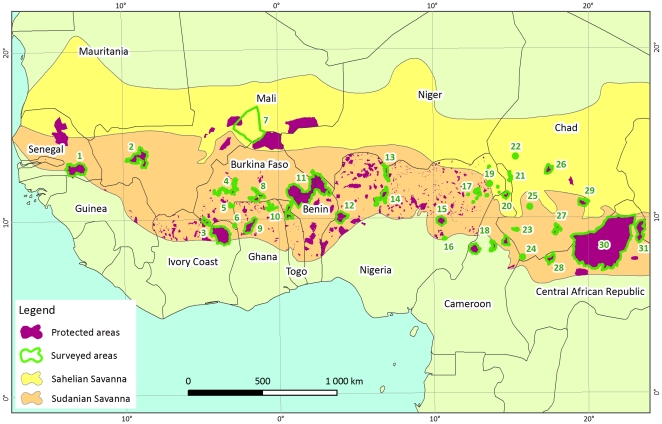
Protected areas surveyed. Protected areas labels (numbers) refer to Table S1.

The study area covers the Sudano-sahelian range occupied by the savannah elephants of West and Central Africa, an area extending over 6000 km from the Atlantic Ocean up to the Red Sea. It covers more than 6,213,000 km^2^ or 21% of the African continent. Savannah elephants once ranged as far as the forests to the south of this ecoregion, but the isolated nature of current protected areas and the loss of forest cover make this unlikely today. The present study covers the following countries: Senegal, Mali, Côte d'Ivoire, Burkina Faso, Ghana, Togo, Benin, Niger, Nigeria, Cameroon, Chad and Central African Republic (Figs. 1 & 2). The total area surveyed in this study was 269,800 km^2^ (Table S1, Fig. 2) or 5% of the Sahelo-Sudanian range, and focused on the areas where elephant populations are mostly likely to persist these last decades. Areas where elephants are widely thought to have been extirpated were not sampled.

**Table 1:** Sudano Sahelian Range's Elephant population numbers according to African Elephant Database standards.

	Definite	Probable	Possible	Speculative
West Africa				
Aerial total count	6346	0	0	0
Aerial sampling count	221	320	453	0
Informed Guess	131	0	0	133
Total West Africa	6698	320	453	133
Central Africa				
Aerial total count	1038	0	0	0
Aerial sampling count	9	59	113	0
Informed Guess	0	0	2000	1710
Total Central Africa	1047	59	2113	1710
Sudano Sahelian range				
Aerial total count	7384	0	0	0
Aerial sampling count	230	379	566	0
Informed Guess	131	0	2000	1843
Total range	7745	379	2566	1843

The Sudanian zone is the more mesic area of this range and extends from Senegal to Ethiopia, covering 3,731,000 km^2^. It receives between 500 and 1400 mm rainfall per annum. The mean annual temperature is between 24 and 28°C. The most common habitats are dry forest, various types of woodland, riparian forest and bush fallows with *Vittelaria paradoxa, Combretum spp. Acacia spp., Anogeissus leiocarpus*, *Afzelia africana, Burkea africana, Isoberlinia doka, Terminalia spp., Pilostigma spp.*, *Balanites aegyptiaca, Detarium microcarpum, Lophira lanceolata, Parkia biglobosa, Prosopis africana, Tamarindus indica* and *Ziziphus spp*. [11].

The relatively xeric Sahel strip extends from the Atlantic Ocean to the Red Sea and covers 2,482,000 km^2^. Rainfall is between 150 and 500 mm per annum. The mean annual temperature is between 26 and 30°C. The most common habitats are Sahel wooded grassland, semi desert grassland and secondary grassland with *Acacia tortilis, A. seyal , Commiphora africana, Balanites aegyptiaca*, with graminea including *Andropogon gayanus, Aristida stipoïdes, Cenchrus biflorus, Schoenefeldia gracilis,* and *Tragus racemosus.* [11].

### Population status and distribution

Elephant distribution and population size were assessed using a variety of survey techniques. Recent surveys, conducted primarily by the authors, were mainly performed using aerial census techniques (described below). Older surveys used aerial census, ground counts, and expert opinion. Data from previous surveys and information used to gauge the over-all status of the region's elephant populations were assessed by literature review and discussion with local managers and researchers. Here, we refer to a “population” as the elephants inhabiting a particular area (such as an ecosystem or protected area).

Expert opinion was relied upon where functioning research or management was active in a given area. For the data presented here, expert opinion typically is used to gauge areas where elephants are extirpated or in extremely low density. Expert opinion on extirpated populations is drawn from non-censusing patrolling, biodiversity inventories and discussion with illegal users of park resources.

#### Recent Aerial total count Surveys

Current numbers of savannah elephants in most sites were estimated from aerial total counts [12]. Total area counts were conducted on 75,272 km^2^ of which 68,226 km^2^ (91%) was surveyed by the first author. Aerial count methodology was conducted as follows: sites were divided into blocks of similar size corresponding to the area that can be covered by a single day survey of 7 to 8 flight hours. To minimize the risk of herd movements from one block to another, which can result in double counting, the largest area possible was covered each day and block boundaries were designed to correspond more or less with drainage basins of major watercourses. Surveys were all carried out at the end of the dry season (April–May) when water was confined to large rivers where most wildlife concentrated. In order to scan the total area of each block, east–west oriented flight lines were generated at one-kilometer intervals. Flight lines extended 2vkm beyond the block limit to overlap neighboring blocks in order to provide insight on how many animals were being missed.

Because of the enormity of the Gourma range (Fig. 2), a different survey plan was instituted. Radio-telemetry data indicated elephants concentrate close to a few permanent lakes at the end of the dry season [I] (Document S1).

Thus counting efforts concentrated around these permanent lakes at that period of the year. Surveys were flown during the cooler hours of the day; starting at 5:45 am local time and lasted for 4 to 5 hours, followed by a refuel and rest stop of 3 hours before afternoon flights.

Cessna 172, 175, 177, 182 and 206 high wing aircraft (Cessna Wichita Kansas USA) with at least 4 seats were used. One to 6 planes were engaged in each survey operation. During the survey, the pilot maintained the altitude appropriate to the conditions (visibility, type of vegetation, etc.). The average height was around 90 m above the ground level. The average flight speed was 160 kph. Pilots followed flight lines on the moving map screen of GPS units.

Each participant was trained on survey techniques [12] for 3 to 5 hours prior to participation. Rear seats observers (RSOs) counted animals on their respective sides and communicated the observation orally to the front seat observer. At the request of the RSOs, the pilot left the flight line and circled to facilitate counting and photography. The front seat observer (FSO) recorded on his data sheet the observations called by the RSO, and the waypoint from a Garmin GPS 72 (Garmin Olathe Kansas USA). The continuous track log of the GPS recorded the flight track. After each flight evening debriefings focused on the identification of possible double counts. A georeferenced database was built for each survey using the observation and GPS data. The location and composition of each elephant herd (number of adults, sub-adults, young and babies) in the GIS identified any remaining double counts.

For large herds (more than 15 elephants), pictures were taken with digital cameras. Each image was projected onto a screen by a LCD projector. Each sub group was circled, tallied and then summed to give the total for the entire herd.

The counts from each block, excluding double-counted herds, were summed to give the estimate for each survey. It is unlikely that all herds were seen, especially along thick forest galleries in the south of the Sudanian range. Therefore, the total counts presented are minimum estimates. They fall in the category of “definite population” (which corresponds to the population estimate from an aerial total count) according to the African Elephant Database standards [6].

#### Older surveys

Historic counts were obtained from IUCN (International Union for Conservation of Nature) African Elephant Database Reports published between 1995 and 2007 [6], [13]–[15], international papers [2]–[4], [16]–[30] and unpublished reports [I-XLV] (Document S1). The quality of data on elephant estimates was evaluated by the standards of the African Elephant Database Reports [6], [15].

The earlier surveys were aerial sample counts [31] and distance sample ground counts [32]. In the early 2000s the common use of the total count method [12] in the range facilitated comparison of estimates. Here we assume that the survey biases are similar from one count to another. The current population size was calculated using the most recent survey results recorded between 2002 and 2009. Additional information regarding elephant status and movements was collected from interviews with wardens, rangers, and communities during field missions between 2002 and 2009 as described previously.

### Elephant population trends

Three approaches were used to calculate changes in population size for areas that were surveyed on multiple occasions. (1) Direct numerical comparisons: for locations where counts could be extrapolated for identical survey areas (or within 90% of the same area), counts were compared directly. (2) Direct numerical comparison from overlapping survey areas: for those areas where agricultural expansion has greatly reduced the size of protected areas or potential area inhabited by elephants, numbers from historic surveys of larger regions are compared directly to recent surveys of smaller areas covering the total remaining range by extracting the data from the overlapping area covered by successive counts. (3) Density comparison: for surveys that covered relatively the same area, but numbers for directly overlapping regions could not be extracted due to unavailable raw data, we compared densities between historic and contemporary counts.

Surveys results were classified in 5-year periods (1960 to 1964, 1965 to 1969 etc.). Estimates for the same population derived from aerial and ground counts were not compared quantitatively. For populations at densities to low to effectively survey (extremely low densities), information on presence or extirpation of elephants was assessed from the expert opinions of the protected area managers. Where there were several estimates from different methods in the same 5-year period, the estimate from the last aerial count within that period was used.

Trends are reported as the percent (positive or negative) change between successive counts using mean estimates of aerial sample count or estimate from total counts. Significance of changes between two estimates from sample counts was assessed using the *d* test [33]. Changes between total and sample counts were considered significant when the total count number was larger or smaller than the confidence interval of the sample count. A change between total counts estimates was considered significant when the difference was greater than 10%.

Sub-regional trends for Central Africa were calculated by summing population estimates (one per population) from 1986–91 and comparing it to the sum of corresponding population estimates from 2005–2010. Similarly, West Africa regional trends were derived by comparing estimates from 1980–83 to those from 2003–07 (Table S2). For this regional trend analysis, compared data were derived from aerial counts, ground counts and guess estimates. Those populations for which historic and recent survey areas differed were not included in this analysis; population specific comparisons for these areas present changes in density and were not conducive for inclusion in regional trend analysis. For the West Africa region, 4.4% of populations were excluded. All populations were used in regional trend analysis for Central Africa. Survey areas differed in historic and recent counts in the Central African Republic (C.A.R.), comparison was limited to overlapping portions of the 1985 and 2005 aerial counts (Table S2) [25] .

For some locations, successive survey reports provided different survey area size for the same study area (e.g Pendjari, Niokolo Koba). In reality the study area didn't change in size. The official area size was used to compare counts between them.

Carcass ratios: 
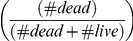
 [34] are assessed to gain insight regarding the role played by illegal killing in the presented population trends.

## Results

### Current Elephant population status

**Figure 3:**
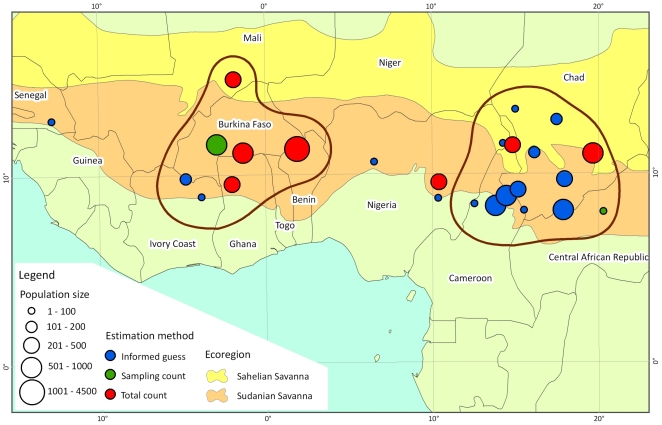
Elephant population's size and distribution in the Sudano Sahelian Range.

The total minimum estimate of definite elephants in the Sudano-Sahelian range is 7,745 (Table 1). West African populations contain 86% of the range's definite numbers (Table 1), with 96% of the known elephant population of West Africa having been surveyed recently by aerial counts (Table S1, Fig. 3). In the last ten years, 4 of 13 populations in Central Africa have been surveyed by aerial counts (Table S1, Fig. 3), representing 10% of the elephant population of Central Africa. Current estimates put 37% of the Sudano-Sahelian range's elephant population in Central Africa, but these estimates are likely high (see discussion below). West Africa is thought to hold at least 63% of the entire Sudano-Sahelian range's elephants.

### Study area population trends

The total regional elephant populations studied declined by 50% in the 15 to 30 years across which surveys were conducted (Tables 2 and 3). Declines were most prevalent in Central Africa, with a known decrease of at least 76% since the late 1980′s. The West Africa region also experienced declines with a minimum 33% decline between 1980–83 and 2003–07. All trends were statistically significant (Tables 2 and 3). Population trends, however, were not uniform across individual populations.

**Table 2:** Central and West African elephant population trends.

Central Africa	85–91		2005–10	
	Estimate	CI95%	Estimate	CI95%
Waza	1071		246	
Zakouma	1040		542	
Bamingui Bangoran	1607	914	708	406
Manovo Gounda	2701	887	74	71
Total CA	6419	1274	1570	412
West Africa	80–83		2003–07	
	Estimate	CI95%	Estimate	CI95%
Mole	*589*	*486*	395	
Gourma	550		344	
Niokolo	50		1	9
Yankari	280		348	
Nazinga	230	280	548	
Po	112	93	64	
Arly Singou	2335	1074	2541	
Pendjari	826	480	869	
W	1331	728	1094	
Comoé	1250	250	10	10
Kainji	1500		0	
Mouhoun	150	180	22	56
Bontioli	100		20	
Total WA	9303	1527	6256	58

Mole elephant population was assumed to be stable since the 80′s

CI95%: 95% Confidence Interval. Elephant population estimates of Central Africa for the periods 85–91 and 2005–10 and West Africa for the periods 80–83 and 2003–07.

**Table 3:** Elephant population trends from 90–91 period to 2003–10 period and *d* test.

Region	80–91		2003–10		trend	*d test*	*P*
	Estimate	CI95%	Estimate	CI95%			
Central Africa	6419	1274	1570	412	−76%	7.2445	*P<0.001*
West Africa	9303	1527	6256	58	−33%	3.9878	*P<0.001*
Total	15821	1989	7826	416	−50%	7.8707	*P<0.001*

**Figure 4:**
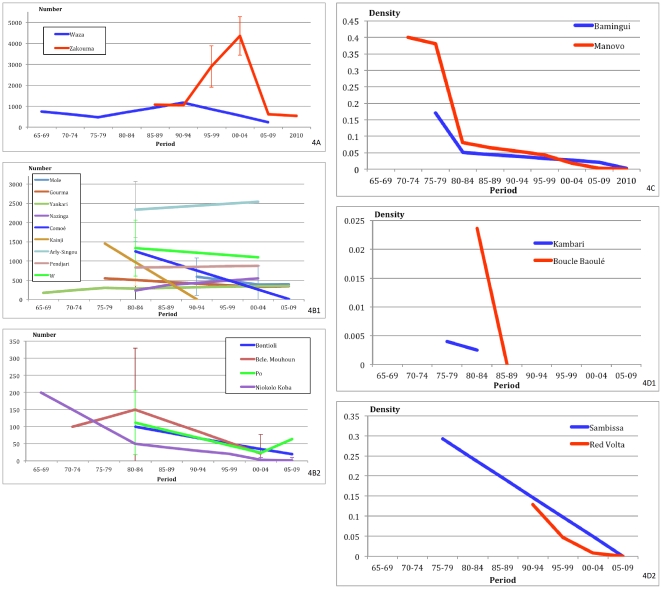
Long term trend of elephant population. Numerical count comparison: A) Central Africa: Waza, Zakouma. B1) West Africa. Populations over 200 elephants: Mole, Gourma, Yankari, Nazinga, Comoé, Kainji, Arly-Singou, Pendjari, W. B2) West Africa. Populations below 200 elephants: Bontioli, Boucle du Mouhoun, Po, Niokolo Koba. Density comparison: Central Africa: C) Bamingui Bangoran, Manovo Gounda St Floris. West Africa: D1) Kambari, Boucle du Baoulé, D2) Sambissa, Red Volta

In West Africa most populations with comparable survey declined significantly during the 1970′–80′s excepted for Nazinga, Arli Singou, and Pendjari, which have increased or remained relatively stable (Fig. 4B1, Table S2). Most small populations (below 200 animals) were extirpated over this time frame or reduced to levels where recovery seems unlikely (Figs 4D1 & 4D2 Table S2), with the exception of Nazinga which increased from the estimated 40 elephants in the late 70′s (Table S2). Population numbering over 300 animals in West Africa were generally stable or increasing. Populations of Boucle du Mouhoun and Po were almost extirpated in the 1990s after a decline of over 85% in 20 years (Fig. 4B2, Table S2). The Po population has increased (+167%) in the last decade (Table S2). After a decline likely induced by drought (elephant poaching is rare in this area), the Gourma elephant population has been stable (Fig. 4B1, Table S2).

West African populations for which sequential surveys were not directly comparable (Niokolo Koba, Bontioli, Red Volta, Comoé Complex, Kainji) are all believed to have been extirpated (or nearly so) with the exception of Yankari (which increased since the 1960′s). The increase in Yankari is believed to result from elephants concentrating in the park as a result of the increasing human pressure in outlying unprotected areas [V] (Document S1).

Central African populations for which comparable surveys were conducted all demonstrate precipitous declines, particularly in the last decade. The population of Zakouma and Waza N.P. increased in the 90s, hypothesized to result from immigration resulting from agricultural expansion and hunting pressure outside the parks, but have declined since to current levels at historical lows (Fig. 4A, Table S2). In northern C.A.R. sustained declines have occurred for the past three decades (Fig. 4C).

Proper surveys are not available for other Central Africa populations, and informed guesses are more than 5 years old [6]. According to guess estimates, Chad shelters the largest number, but recent civil disturbances in Chad and dramatic declines in Zakouma NP, the only monitored Chadian population, suggest the guess estimates presented in Table S1 are suspect. Similarly, the guess estimates in Table S1 for the northern protected areas of Cameroon are dubious given popular reports of the impact of illegal ivory trade and the conflicts in neighboring Chad and CAR. Double counting of transboundary populations (Nigeria, Cameroon and Chad) is another concern of guess estimates. Collared elephant in Waza NP (Cameroon) have been observed to use multiple areas, crossing the Nigerian border [35] (Fig. 3).

### Elephant population distribution

Currently, most Western and Central African savannah elephant populations are scattered and isolated (Fig. 3). The surveyed protected areas can be grouped in two major complexes of protected areas, within which potentially interacting elephant populations are located. In West Africa, 7 of 12 populations representing 89% of the West African population (6690 individuals) live within 109 km (median) (range: 90 to 396 km) from each other, and are part of a western pool concentrated in a radius of 425 km around Burkina Faso. This western pool includes populations from Benin, Burkina Faso, Côte d'Ivoire, Ghana, Mali, and Niger (Fig. 3). In Central Africa, 7 of 13 populations representing 84% of the Central African population (3773 individuals) live within 230 km (median) (range: 33 to 341 km) from each other (Table S1). This eastern pool in Nigeria-Cameroon and Chad is concentrated more or less within the Lake Chad Basin (Fig. 3). The shortest distance between known populations in the two pools is about 830 km, a gap mainly covering Nigeria, the most densely human populated country of Africa. Therefore, it is unlikely that elephants currently inhabit areas within this gap out of the isolated protected areas where elephants still remain (e.g. Yankari).

### Carcass record

**Figure 5:**
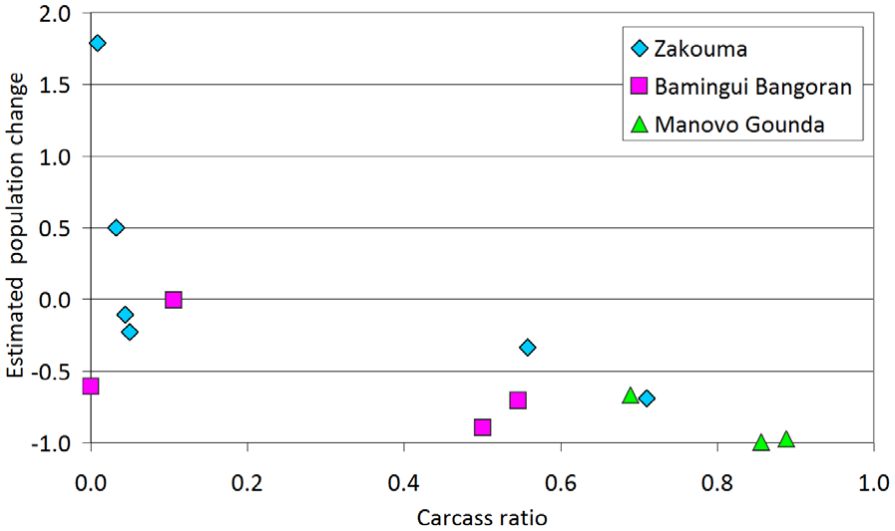
Carcass ratio versus estimated population change in Zakouma, Bamingui Bangoran and Manovo Gounda Saint Floris.

Determining causes of declines is difficult. Analysis of the carcass ratio in Zakouma, Bamingui Bangoran, and Manovo Gounda Saint Floris NPs in Central Africa demonstrates the impact of illegal killing in this region, where precipitous declines are strongly correlated with rising carcass ratios (Fig. 5). As these populations collapsed, the number of recorded carcasses exceeds the number of live elephants (Fig. 5, Table S2). In contrast, the most recent West African counts show small carcass ratios (Table S2).

## Discussion

### Drivers of current elephant status

Across the Sudano-Sahelian range, elephant populations are typically declining, but trends over the past three decades vary by location. Central Africa demonstrated the greatest declines [25,26,36,IV,XI] (Document S1) most likely resulting from illegal hunting for ivory (Fig. 5). In Central Africa, the lack of law enforcement personnel and failure in law enforcement organization and implementation have allowed the proliferation of illegal activities that threaten elephants and wildlife in general [25,XI] (Document S1). For example, the law enforcement staff for the Programme de Développement de la Région Nord (PDRN) and successive projects in northern CAR has been at less than 10% of the required level since their inception [25]. In West Africa, with the exception of Côte d'Ivoire and Nigeria which lost more than 1000 elephants in recent decades probably due to poaching [20], elephant poaching is currently sporadic most likely because it is difficult to poach discreetly. Major illegal ivory markets in Côte d'Ivoire, Nigeria, Sudan and Cameroon [37] continue to be a source of pressure on elephant populations in the region. Unfortunately, much of the ivory in these markets originates in Central Africa [37] with Côte d'Ivoire and Nigeria trading annually a volume of illegal ivory exceeding that of their respective current live elephant populations.

Land use change has also been a dominant factor shaping population change and distributions of the region's elephants in the last 40 years. One of the primary drivers of land use change in the region is climate variation and change. A drought that hit the Sudano Sahelian range in the early 1970s, with major decreases in rainfall [38] and surface water (Lake Chad lost 70% of its surface area during this period [8]), had considerable impacts on livelihoods across the range spurring marked changes in crop production and food security policies [39]. Agricultural expansion intensified, in part due to a need for new land for cultivation to replace exhausted soils [40]. In addition, cattle transhumance, originally restricted to the Sahelian range, extended southward as herders searched for better pastures and water during the dry season [38]. Transhumant herders frequently enter protected areas to access pastures and water for their cattle [41]–[44], since cultivators dominate unprotected lands.

Recorded declines of elephants in some areas experiencing land use changes may be a function of emigration to safer areas as much as mortality. Specifically, increases in Nazinga and the WAP ecosystem are thought to reflect immigration resulting from land use change in outlying areas [19], [23]. In most areas, remaining elephants are now isolated in a few protected areas [3], [4], [45] surrounded by agriculture (Figs. 2 & 3). This spatial constriction bounded by increasing human activities results in human elephant conflicts, typically amplifying human driven elephant mortality [3], [46].

### Spatial distribution and conservation priorities

Results from this study demonstrate elephants in the Sudano-Sahelian range are now limited to two zones, and the populations within each zone are increasingly isolated due to changes in land use. Episodic contact among elephant populations occurs, but this connectivity is being increasingly severed particularly in West Africa [4], [22]. While reducing human impacts (e.g. illegal killing) is critical to the persistance of elephants across this range, conserving links and establishing corridors between the largest populations and largest blocks of protected areas should be a primary management objective particularly in West Africa where poaching is better controlled. Maintaining contact is particularly critical to the long term viability of remaining small populations that may not be viable in isolation. In addition, linking protected areas across borders is particularly important given the transboundary nature of many of these populations' historic range. Cross-border linkages also have the advantage of allowing movement to or from neighboring countries in time of war or civil troubles (e.g. during civil disturbances in Togo during the early 1990s more than 100 elephants found refuge in Burkina Faso and Benin [19] and Ghana [46]).

**Figure 6:**
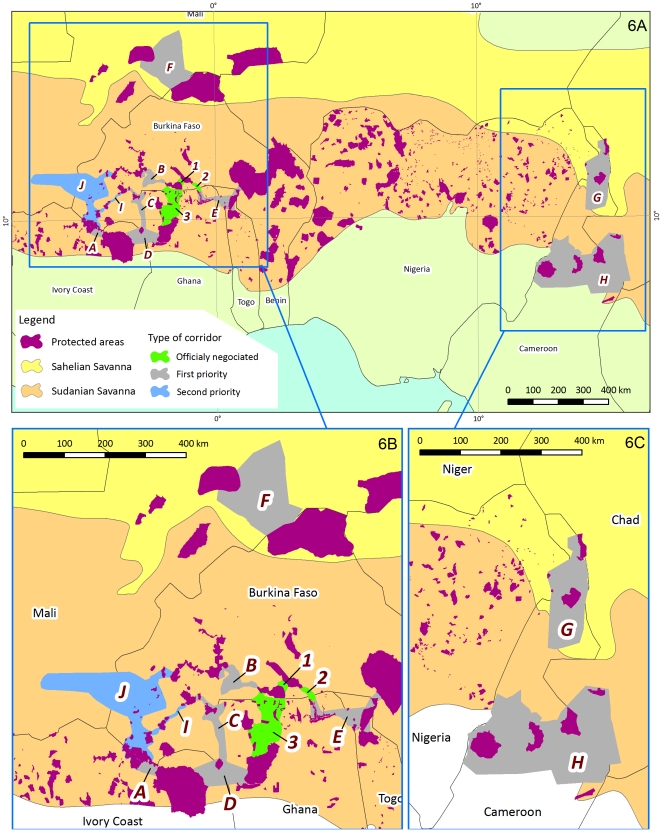
Corridors. A) Current and proposed corridors in the Sudano Sahelian Range. Corridors labels (letters or figures) refer to Table 4. B, C) Zoom on West and Central Africa parts of the study area.

We propose a list of corridors that maintain critical links within these two zones (Fig. 6, Table 4). The establishment of these corridors will necessitate overcoming substantial obstacles, including biological and political issues [47], [48]. Many areas between protected areas are currently cultivated, as such the feasibility of a corridor will depend on the willingness of local people to pursue land use changes consistant with a corridor. Derived benefits from such a conversion will be critical to the long term success of corridor establishment. Projects focused on community participation with private operator (e.g. hunting outfitter) involvement appear to be the best model in respect to elephants [21], [49]. Around the Cameroonian parks where elephants are still common, the area between Faro, Benoué, Bouba Ndjida, Gagal Yapala and Larmanaye which holds 26 hunting areas is a high potential site for a landscape scale management project of this nature (Fig. 6).

**Table 4:** Proposed corridors.

Label	Corridors type and name	Comments
	Current corridors	
1	Po-Nazinga	Elephants use this corridor regularly. This corridor was officially opened in 2006.
2	Po-Red Volta	Opened in 2006. Elephants use it regularly. These last years they seems not to cross the Ghanaian border.
3	Nazinga Mole	Elephant use to roam between Nazinga and Mole during the 1980′s. The corridor was opened in 2008.
	First priority corridor	
A	Comoé Complex	This corridor was used recently during the Côte d'Ivoire civil war. 130 elephants migrate from Côte d'ivoire to Burkina Faso.
B	Nazinga-Boucle du Mouhoun	Used sometimes by elephants during the rainy season. Community took the initiative to build the corridor. Today they manage an area slightly larger than 1000 km^2^ and cover about the half of the distance.
C	Bontioli-Koulbi	The feasibility and negotiation of this corridor is managed by the Progeref project and should be extended into Ghana.
D	Comoé-Mole	There is currently no connection between the two parks. A corridor should be opened between Mole NP and Comoé NP through Koulbi Forest Reserve in Burkina Faso.
E	PONASI-WAPOK	Elephants used to roam this area up to early 2000′s. Elephants seems have disappeared from this area today. Currently no contact between the two larger West African elephant's populations seems to exist any more. High human population density occurs between the two complexes. The corridor should enlarge the current Red Volta complex that is too narrow. The corridor should merge the northern Ghana and Togo. The corridor feasibility should be evaluated.
F	Mali-Burkina Range (Gourma)	The last Sahelian elephant's range must be secured despite the traditional respect of the local people. This remain one of the most vulnerable population of West Africa [3]. It suffers from the direct and indirect consequences of the drought that started in the early 1970s and from lack of field management. The competition for water has reached an intolerable level both for people and elephants. A policy and management plan is needed to segregate elephant movements from human activities and to guarantee water for elephants [24], [48].
G	Waza-Kalamaloué	Elephant use to roam between the two areas but need better protection. The area between the two protected areas is also very human populated.
H	Faro-Benoué-Bouba Njida	Hunting areas, surrounding national parks, mainly covers this area. Therefore the corridor could be It maybe harbors the larger savannah's elephant population of Cameroon.
	Second priority corridor	
I	Comoé-Nabéré	This is currently used as an occasional corridor by elephants
J	Comoé-Boucle du Mouhoun	This is currently used as an occasional corridor by elephants coming from Mali and Burkina

Current corridors: official corridors agreed both by authorities and communities. First priority corridors: top priorities for conservation at the national or regional scale linking the main elephant populations. Second priority corridors: secondary priorities for conservation at the national or regional scale linking small elephant populations.

In addition to corridor establishment, transboundary management strategies are needed to coordinate activities across the *de facto* elephant range. For instance, the elephants of Northern Cameroon, Nigeria and Chad are known to be part of a single complex [35], therefore a simultaneous survey is required to count the populations of Waza complex and those of the Chad Basin in Nigeria and Massenya Madjafa in Chad. Another simultaneous survey should evaluate the links between the Faro, Benoué, Bouba Ndjida, Gagal Yapala and Larmanaye area. Information on the status of these transboundary parks and connectivity of these landscapes is essential prior to the implementation of management actions.

Despite considerable human and financial efforts in the most renowned savannah parks of Central Africa, elephant conservation has failed (Figs. 4A & 4C), with the failure especially marked in less stable countries (Chad, CAR) where the maintenance of savannah elephant populations is looking unlikely. Where repeated survey data is available, counts demonstrate that Sudano-Sahelian elephants have been declining for the past four decades (Figs. 4A to 4D2). While populations are increasingly isolated, community and privately managed areas can improve connectivity. To conserve the last remaining populations in northern Africa, the common efforts of all elephant range states, international institutions and NGOs are required.

## Supporting Information

Table S1 **Current Sudano Sahelian Range's Elephant populations: site number (see** **Fig. 2** **), area name, year of count, area size (km^2^), results from aerial total count (TC), aerial sampling count (SC), ground count (GC), informed guess (IG): estimate (Est), confidence interval (CI), summary of estimations (Summary), density (animal/km^2^), proportion of total population (% of tot pop), distance (Km) to the edge of the nearest viable population (>300 individuals).** Added citations: 50. UICN BRAO 2008. Evaluation de l'efficacité de gestion des aires protégées : Parcs et réserves du Mali. UICN 51. Yer M (2006) Plus de 100 pachydermes à Koumadara dans le Houet. Journal Sidwaya June 22, 2006. Burkina Faso.
(XLS)

Table S2 **Four decades of live elephant and carcass number or density comparisons in West and Central African savannahs.**
(XLS)

Document S1 **Unpublished reports.**
(DOC)
