# Supplementary material for: Will Elephants Soon Disappear from West African Savannahs?
Source: PLoS One. 2011 Jun 22;6(6):e20619. doi: 10.1371/journal.pone.0020619 (PMC3120750; doi:10.1371/journal.pone.0020619)
Supplement: Document S1 — Unpublished reports. (DOC) [file pone.0020619.s003.doc]

Unpublished reports

1. Blake S, Bouché Ph, Rasmussen H, Orlando A and Douglas-Hamilton I (2003) The last Sahelian Elephants. Ranging behavior, population status and recent history of the deserts elephants of Mali. Save the Elephant.
2. Bouché Ph, Lungren CG, Hien B and Omondi P (2004) Recensement aérien total de l’Ecosystème W-Arly-Pendjari-Oti-Mandouri-Kéran (WAPOK). CITES-MIKE, ECOPAS, PAUCOF, Benin, Burkina Faso, Niger, Togo.

Bouché Ph, Lungren CG and Hien B (2004) Recensement aérien total de l’Ecosystème Po Nazinga Sissili (PONASI). CITES-MIKE. Ministère de l’Environnement et du Cadre de Vie. Burkina Faso.

1. Bouché Ph (2010) Inventaire aérien 2010 des grands mammifères dans le Nord de la République Centrafricaine. Composante ZCV. ECOFAC. CITES-MIKE. RCA.
2. Omondi P, Mayienda R, Mshelbwala JH and Massalatchi MS (2006) Total Aerial Count of Elephants, Buffaloes, Roan Antelope and other Wildlife Species in Yankari Ecosystem, Nigeria. CITES MIKE, EU.
3. Omondi P, Mayienda R and Tchamba M (2007) Total Aerial Count of Elephants, Giraffes, Roan Antelopes and other Wildlife Species and Ostrich in Waza National Park, Cameroon. WWF, USFWS.
4. Mackie C (2002) Recensement aérien de la grande faune du Parc national de Zakouma. Mee/Dpfpn/Curess, Tchad
5. Fay JM, Dolmia NM, Boulanodji E, Ndoninga A, Guggemos C et Poilecot P (2005) Comptage aérien total de la grande faune du Parc national de Zakouma. Mee/Curess, Tchad
6. Fay JM, N’Gakoutou EB, Taloua N, Poilecot P et Ndoninga A (2006). Dénombrement aérien total des grands mammifères et de l’autruche du Parc national de Zakouma, Tchad. Mee/Dcfap/Curess, Tchad
7. Poilecot P (2008) Comptage aérien des grands mammifères (avril 2008) et planification d’une stratégie de suivi écologique au Parc national de Zakouma (Tchad). Rapport final. Agri-for/Eco Consult/Gopa/ Curess.
8. Potgieter D, Taloua N, Djimet B and Fay M (2009) Dry season aerial total count, Zakouma national Park, Chad. WCS/UE/Curess
9. Spinage CA, Loevinsohn ME and Ndoute J (1977) Etudes additionnelles du Parc National Bamingui Bangoran. CAF/72/010. Document de travail 8. FAO. Rome
10. Douglas Hamilton I, Froment JM, Doungoube G and Root J (1985) Recensement aérien de la faune dans la zone Nord de la République Centrafricaine. Aménagement de la faune. République Centrafricaine. FAO. FO CAF/78/006. Document de travail 5.
11. PDRN (1998) Situation de la faune de grande taille dans les zones protégées du Nord de la RCA. 22-69 *in*. Rapport annuel. Unpublished report. UE/FED. NORCADEV, RCA
12. Renaud P-C (2005) Recensement aérien de la faune dans les préfectures de la région Nord de la République Centrafricaine. Rapport. ECOFAC III
13. Loevinsohn ME, Spinage CA, Ndoute J (1978) Analyse des résultats de survol aérien 1978. CAF/72/010. Document de travail 10. FAO. Rome
14. Bousquet B (1982) Inventaire des ressources en faune sauvage et étude économique sur son utilisation en zone rurale. Résultats des inventaires aériens de la faune. Haute Volta, FAO FO : DP/UPV/78/008 ; Document de terrain N°6 Rome.
15. Belemsobgo U (2002) Résultats de l'analyse de l'inventaire aérien de la grande faune et du bétail dans le complexe des aires protégées de la boucle du Mouhoun en avril 2002 (Unpublished report). Ouagadougou: Direction des Parcs Nationaux, Réserves de Faune et des Chasses.
16. Bouché Ph (2005) Inventaire aérien de la faune dans les sites du PAGEN : Forêt Classée et Réserve Partielle de Faune de Comoé-Léraba ; Forêts Classées de Boulon et de Koflandé ; Réserve de la Biosphère de la Mare aux Hippopotames , Parc National Kaboré Tambi. Ministère de l’Environnement et du Cadre de Vie. Burkina Faso.
17. Bouché Ph (2007) Inventaire de la faune dans la forêt Classée de Koulbi et dans les Réserves de Bontioli. SOCREGE, PROGEREF, Ministère de l’Environnement Burkina Faso
18. Sam MK, Okoumassou K and Barnes RFW (1996) A preliminary survey of the elephants of north-eastern Ghana and northern Togo. unpublished report, Wildlife Department, Accra and Direction des Parcs Nationaux, des Reserves de Faune et de Chasses, Lomé.
19. Sam MK (1998) An assessment of crop damage by elephants in the Red Volta Area of Ghana
20. Adjewodah P (2004) Habitat status, population and distribution of the African savanna elephant (*Loxodonta africana*) in Northeastern Ghana. NCRC, IUCN AfESG Project SG0203 Final report
21. Bouché Ph (2006) Northern Ghana Wildlife Survey. IUCN, NSBCP.
22. Wilson VJ (1993) A zoological survey of Mole National Park. North western Ghana. Part I. Large Mammals. Forest Resource Management Programme. Game and Wildlife Dept/IUCN Project 9786 Accra Ghana
23. Bouché Ph (2002) Elephant survey of Mole National Park. Ghana. March 2002. Report of the aerial survey. CITES-MIKE.
24. Mackie C (2004) Mole aerial survey. Wildlife Division, IUCN.
25. Douglas-Hamilton I (1979) The African elephant action plan. Typescript Report to IUCN
26. La Marche B (1978) Les éléphants au Mali: le Gourma et l’est. Bamako.
27. Jachmann H (1991) Current status of the Gourma elephants in Mali: a proposal for an integrated resource management project. IUCN, Gland, Switzerland.
28. Bouché Ph (2007) Dénombrement des éléphants du Gourma. PCVBGE, AFD, UICN.
29. Mauvais G (2002) Dénombrement aérien de la moyenne et grande faune et localisation des points d'eau, Parc National de Niokolo-Koba, Saison 2001/2002. Dakar: Direction des Parcs Nationaux du Sénégal.
30. Renaud PC, Gueye MB, Hejcmanová P, Antoninova M and Samb M (2006) Inventaire aérien et terrestre de la faune et relevé des pressions au Parc National du Niokolo Koba. Dakar: Ministère de l'Environnement et de la Protection de la Nature and African Parks Foundation.
31. Cornélis D (2000) Analyse du monitoring écologique et cynégétique des populations des principaux ongulés aux Ranch de Gibier de Nazinga (Burkina Faso). Fac. Universitaire des Sc. Agro. Gembloux
32. Bousquet B and Szaniawski A (1981). Résultats des inventaires aériens des grands mammifères dans la région « Pendjari Mekrou ». Bénin et Haute-Volta, FAO FO : DP/UPV/78/008 DP/BEN/77/011; Document de terrain N°4. Version non officielle
33. Marchand F, Lacroix F, Pasquet H, Sebogo L and Lamarque F (1993) Projet :« Sauvegarde des Eléphants du Burkina Faso ». Rapport Final. Ministère de l’Environnement et du Tourisme Burkina Faso, Ministère de la Coopération, République Française
34. Barry I and Chardonnet B (1998) Recensement aérien de la faune de l’Unité de Conservation d’Arly. Résultats et commentaires. Ministère de l’Environnement et de l’Eau. Burkina Faso.
35. Chardonnet B, Rouamba P, Barry I, Ouédraogo A and Nacoulma P (1999) Suivi écologique aérien des aires classées des bassins de l’Arly et du Singou. Ministère de l’Environnement et de l’Eau. Burkina Faso.
36. Bouché Ph, Heymans J-C, Lungren CG and Ouedraogo LK (2000) Recensement des animaux sauvages dans les concessions de faune de l’Est. Ministère de l’Environnement et de l’Eau. Burkina Faso. UICN
37. Rouamba P and Hien B (2002) Recensement aérien de la faune sauvage dans la Réserve de la Biosphère de la Pendjari. Ministère du Développement Rural, CENAGREF. Bénin.
38. Delvingt W (1987) Programme d’aménagement des Parcs Nationaux et de protection de l’environnement. Rapport sur l’Evolution des populations de grands mammifères dans le Parc National et la Zone cynégétique de la Pendjari. Ministère du Développement Rural et de l’Action Coopérative. République Populaire du Bénin*.*
39. Sinsin B (2001) Dénombrement de la faune dans la Réserve de la Biosphère de la Pendjari Avril 2001. PCGPN. CENAGREF, GTZ.
40. Sinsin B, Akpona A and Ahokope E (2006) Dénombrement aérien de la faune dans la Réserve de Biosphère de la Pendjari (Rapport provisoire). Cotonou: Université d’Abomey-Calavi, GFA.
41. Frame GW, Lungren CG, Herbison-Frame L and Lungren RF (1991) Estimations des populations des grands mammifères lors d’un recensement aérien en mars 1991 au Parc National du W Burkina Faso. ADEFA et Direction Provinciale MET, Burkina Faso.
42. Rouamba P and Hien B (2002) Recensement aérien de la faune sauvage dans l’Ecosystème le Parc transfrontalier du « W ». République du Bénin, Burkina Faso, République du Niger Programme Régional Parc – W (ECOPAS). FED
